# An Intelligent Online Drunk Driving Detection System Based on Multi-Sensor Fusion Technology

**DOI:** 10.3390/s22218460

**Published:** 2022-11-03

**Authors:** Juan Liu, Yang Luo, Liang Ge, Wen Zeng, Ziyang Rao, Xiaoting Xiao

**Affiliations:** 1 School of Electric and Information, Southwest Petroleum University, Chengdu 610500, China; 2 School of Mechatronic Engineering, Southwest Petroleum University, Chengdu 610500, China; 3 School of Materials Science and Engineering, Chongqing University, Chongqing 400044, China; 4 State Key Laboratory of Power Transmission Equipment & System Security and New Technology, Chongqing University, Chongqing 400044, China

**Keywords:** drunk driving test, sensor array, data fusion, intelligent detection, alcohol ignition interlock

## Abstract

Since drunk driving poses a significant threat to road traffic safety, there is an increasing demand for the performance and dependability of online drunk driving detection devices for automobiles. However, the majority of current detection devices only contain a single sensor, resulting in a low degree of detection accuracy, erroneous judgments, and car locking. In order to solve the problem, this study firstly designed a sensor array based on the gas diffusion model and the characteristics of a car steering wheel. Secondly, the data fusion algorithm is proposed according to the data characteristics of the sensor array on the steering wheel. The support matrix is used to improve the data consistency of the single sensor data, and then the adaptive weighted fusion algorithm is used for multiple sensors. Finally, in order to verify the reliability of the system, an online intelligent detection device for drunk driving based on multi-sensor fusion was developed, and three people using different combinations of drunk driving simulation experiments were conducted. According to the test results, a drunk person in the passenger seat will not cause the system to make a drunk driving determination. When more than 50 mL of alcohol is consumed and the driver is seated in the driver’s seat, the online intelligent detection of drunk driving can accurately identify drunk driving, and the car will lock itself as soon as a real-time online voice prompt is heard. This study enhances and complements theories relating to data fusion for online automobile drunk driving detection, allowing for the online identification of drivers who have been drinking and the locking of their vehicles to prevent drunk driving. It provides technical support for enhancing the accuracy of online systems that detect drunk driving in automobiles.

## 1. Introduction

As the number of motor vehicles in countries around the world continues to rise, the problem of drunk driving affecting road safety becomes increasingly severe. Intoxicated drivers cause 4.13-times as many traffic accidents as sober drivers [1,2,3], and drunk driving has become a major component of criminal activity. Using Chinese public security department data as an example, more than 1.74 million people were prosecuted for criminal offenses in 2021, of which more than 350,000 were prosecuted for dangerous driving, accounting for 20% of all criminal offenses and ranking first [4]. The control of drunk driving is entirely dependent on the traffic control department, which generally conducts spot checks on vehicles and can only be applied to some vehicles [5]. However, drunk driving is characterized by randomness and dispersion; therefore, the aforementioned methods have obvious limitations. If the online detection system for drunk driving can be installed in vehicles, dangerous driving will be drastically reduced, and safety will be ensured.

In the 1960s, nations across the globe began to develop anti-drunk driving systems. The most prevalent anti-drunk driving technology involves the relevant government personnel and requires blowing air or drawing blood to detect the driver’s blood alcohol content. This method has a low level of automation and is susceptible to false detection and detection omissions [6]. Angel [7] is a new type of on-board alcohol detection instrument developed by Italian researchers in 2009. When the driver’s breath alcohol concentration reaches a predetermined threshold, the system issues a warning and prevents the vehicle from starting. In 2012, TruTouch, a technology company based in Albuquerque, New Mexico, United States, applied optical detection to alcohol detection technology and invented an infrared alcohol detector [8] that can determine whether alcohol is present in the human body based on the amount of infrared light reflected. In 2014, the National Highway Traffic Safety Administration (NHTSA) created a new type of driving technology [9] to combat drunk driving. This technology detected the driver’s blood and breath alcohol concentration. Taking into account the current situation, the majority of studies on anti-drunk driving system technology employ single-sensor technology, which entails the use of a single sensor to measure the same object, and indicates drunk driving based on the measurement results. It is easy for it to form erroneous judgments and cause false alarms, which negatively impacts the driver’s ability to operate normally.

In recent years, multi-sensor data fusion technology has been developed to compensate for the lack of reliability and measurement precision of single-sensor, anti-drunk driving technology. Its defining characteristics include utilizing multiple sensors to measure the same object in order to obtain the multi-source information about the object and making full use of the redundancy and complementarity of multi-source information in order to form a more reliable judgment of the surrounding environment [10,11]. Additionally, it can maintain accuracy in the event of a poor surrounding environment or equipment failure. Shifat [12] utilizes multi-sensor data fusion technology to effectively detect motor running state, which can detect and classify a variety of fault characteristics and improve the accuracy of fault diagnosis. Zuo [13] utilized multi-sensor data fusion technology to collect various underground mobile device detection data in real time, allowing the data management center to reflect the actual monitoring information of underground mobile devices. Liu [14] utilized multi-sensor data fusion technology to eliminate measurement errors for the indoor positioning of smart phones, a technique that is highly stable and robust. The average positioning error is reduced by 37.4–67.6% when compared to the classical extended Kalman filter method. However, no research has been conducted on multi-sensor data fusion technology to achieve the online intelligent detection of automobile drunk driving and improve the reliability and precision of drunk driving detection results.

To sum up, this study carries out research on multi-sensor data fusion technology for automobile drunk driving detection. Firstly, the sensor array is designed based on the gas diffusion model and the characteristics of the car steering wheel are considered. Secondly, the data fusion algorithm model is proposed according to the data characteristics of the sensor array on the steering wheel. Finally, in order to verify the reliability of the system, an online intelligent detection device for drunk driving based on multi-sensor fusion is developed, and different combinations of drunk driving simulation experiments are conducted using three participants.

## 2. Research on Sensor Array for Drunk Driving Based on Gas Diffusion Model

**Figure 1:**
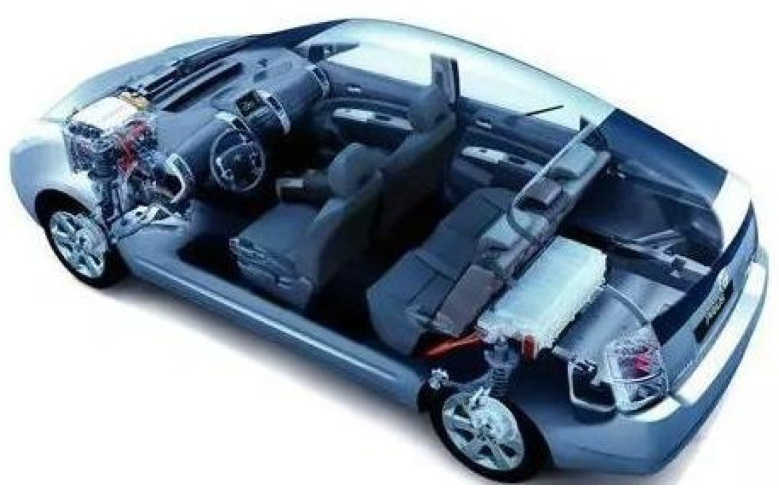
Interior space of the car.

As shown in Figure 1, a car interior space mainly includes the driver’s seat, the co-pilot seat, and the back seat. In order to realize the intelligent online detection of drunk driving, we need to implement accurate alcohol detection to the driver’s seat and lock the car, while avoiding any reaction to the backseat passengers’ and the co-pilot’s alcohol level. Gas sensors can convert chemical signals into easy-to-handle signals to realize the detection and identification of drivers’ exhaled gas after drinking [15]. The selection of gas sensors affects the final detection results [16]. The typical volatile metabolite in drivers’ breath after drinking is ethanol [17]. An MQ3 gas-sensitive sensor can have high sensitivity and good selectivity to ethanol vapor [18], therefore it is selected as the unit of sensor array.

In order to have accurate drunk driving detection, it is necessary to properly select the position of the drunk driving detection sensor according to the location and diffusion process of the ethanol emission source in the car. In the existing research on gas diffusion in a fixed confined space, the widely used gas diffusion models include the Gaussian diffusion model and the gas turbulence diffusion model. Since the Gaussian model does not take the influence of gravity on diffusion into account, it is generally suitable for the leakage and diffusion of light gases with a smaller density than air. However, the density of ethanol is higher than air, therefore the turbulent diffusion model of gas is more suitable [19].

The turbulent gas diffusion model is a static model based on the turbulent diffusion theory. Turbulent diffusion obeys statistical laws, similar to molecular diffusion. Combined with the interior environment of drunk driving detection, the gas diffusion model in the windless environment is discussed. Suppose that, in an infinitely open environment, the gas from the leaking source diffuses slowly around with a constant diffusivity. According to Fick’s law, the diffusion flux $\vec{f}$ through a unit cross-sectional area perpendicular to the direction of diffusion in unit time is proportional to the concentration gradient at this cross-section, and the diffusion direction is the opposite direction of the concentration gradient. The change rate of concentration *C* with time is equal to the negative value of the change rate of the diffusion flux with distance at this point, namely:

(1) $$\left\{ \begin{array}{l} \vec{f}=-k\nabla C\\ \frac{\partial C}{\partial t}=-\nabla \vec{f} \end{array}\right.$$

where *C* (unit: mg/m^3^) is the gas diffusion concentration at position (*x*, *y*, *z*) at time *t*, $\vec{f}$ is the diffusion flux in mg/m^3^, *k* is the diffusion coefficient in m^2^/s, ∂/∂t means the partial derivatives with respect to *t*, and ∇ is the gradient. The diffusion Equation (2) can be obtained from Equation (1):

(2) $$\frac{\partial C}{\partial t}=k\nabla^{2}C$$

where $\nabla^{2}C=\frac{\partial^{2}C}{\partial x^{2}}+\frac{\partial^{2}C}{\partial y^{2}}+\frac{\partial^{2}C}{\partial z^{2}}$.

Given that the gas leakage point at time *t*_0_ (*x*_0_, *y*_0_, *z*_0_) diffuses around at the release rate (also known as source intensity) *Q*, the diffusion concentration can be given by:

(3) $$C(x,y,z,t)=\frac{Q}{4\pi kd}erfc(\frac{d}{2\sqrt{k(t-t_{0})}})$$

where $erfc(x)=(2/\sqrt{\pi })\int_{x}^{\infty }e^{-y^{2}}dy$ is the error compensation function, and *d* is the distance between (*x*, *y*, *z*) and (*x*_0_, *y*_0_, *z*_0_). When actual monitoring is conducted, each monitoring process is often regarded as an equilibrium state, that is, when *t* approaches infinity, the expression is as follows:

(4) $$C(x,y,z)=\frac{Q}{4\pi kd}$$

**Figure 2:**
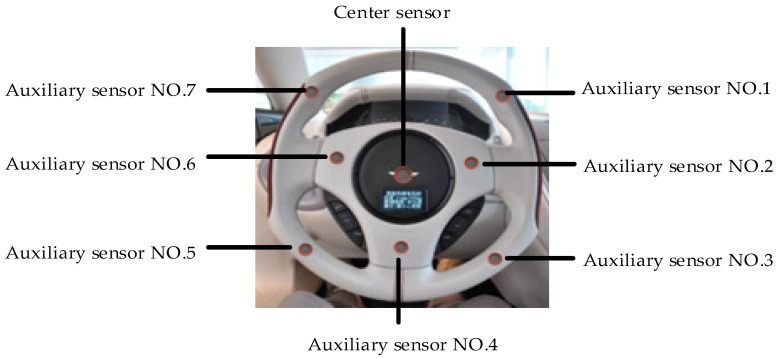
Physical view of sensor array.

According to Equation (4), in a windless environment, the closer the sensor is to the release source, the greater the concentration of the gas is. The interior space of a car can be viewed as a small airtight space without wind. After the driver enters the cab, the breathing gas is closest to the steering wheel. In order to measure the diffusion of ethanol in the car following the consumption of alcohol by the driver, we use the steering wheel in the cab as the measurement point, as shown in Figure 2. The following three cases are discussed in accordance with Equation (4), and detailed information is provided in Table 1.

As shown in Figure 2, a new sensor array is designed primarily for the steering wheel of the cab based on the simple logic of determining the diffusion of ethanol in the automobile. The sensor located in the center of the steering wheel is closest to the gas source when the driver is under the influence of alcohol. Seven auxiliary sensors are located a short distance from the gas source. According to Table 1, it is also necessary to compare measurement data to determine whether or not the intoxicated individual is a driver. The sensor array data fusion algorithm model based on support degree and adaptive weighting is proposed for the fusion processing of multiple groups of measurement signal sequences in order to reduce the random measurement error. This model can be used to determine whether the driver has consumed alcohol and the extent of their drunkenness.

## 3. Model of Data Fusion Algorithm of Drunk Driving Detection with Sensor Array

The first step of the online intelligence detection of drunk driving is to determine whether or not the drinker in the car is the driver. If the individual is a driver, the sensor data should be combined to determine the level of drunkenness. Based on this, the judgment rules for drunk driving are divided into two layers. The first layer detects whether the driver has consumed alcohol. Otherwise, the car will start normally. If yes, it will proceed to the next level of evaluation. The second layer uses the support matrix and adaptive weighted sensor array data fusion results to determine the level of drunkenness, provide a voice prompt, and lock the car.

The first layer of judgment rules combine the bounding sensor matrix to identify the drinking status of the driver. Theoretically, when the driver sits in front of the steering wheel after drinking, the respiration rate received by the central sensor must be greater than that of other sensors in the outer-ring. The mathematical model is shown as follows:

(5) $$\frac{\sum X_{0}\left[10\right]}{10}>\frac{\sum X_{1}\left[10\right]+\sum X_{2}\left[10\right]+\sum X_{3}\left[10\right]+\sum X_{4}\left[10\right]+\sum X_{5}\left[10\right]+\sum X_{6}\left[10\right]+\sum X_{7}\left[10\right]}{70}$$

where *X*_0_ and *X*_n_ (n = 1, 2, ⋯7) represent 10 gas data points collected by the central sensor and the auxiliary sensors within time *t*, respectively. If the data group sampled for 10 cycles does not conform to the relation of Equation (5), the system will take shielding measures on the sampling result and return to wait status for next sampling signal. The first layer of judgment makes sure that the presence of the ethanol source around the steering wheel from people in the non-driver seats will not produce a voice prompt and lock the car, that is, by comparing measurement data to identify non-driver drinking, and to ensure normal start of the car.

**Figure 3:**
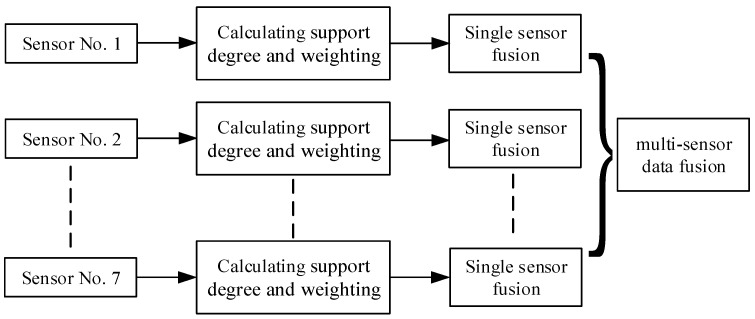
Structural framework of sensor array data fusion.

If the data group of 10 cyclic sampling conforms to the relation of Equation (5), it will lead to the second layer of judgment. The data fusion technology based on the support matrix and the adaptive weighted fusion algorithm is used to process sensor data to improve the reliability of detection and reduce the influence of noise interference [20,21,22]. Firstly, the support matrix is established for the data collected by a single sensor within time t, and the integrative supportability and weighting factor of each measurement data are calculated. Then, based on the data fusion of a single sensor, the measurement data array is fused, and the adaptive weighted equation is established to fuse the array of the measurement signals of the sensor array. The fusion value with the highest support relative to all the measurement values is obtained. The block diagram of the data fusion algorithm is shown in Figure 3.

Single-sensor data fusion is intended to remove deviations caused by the external environment or by noise from measured data. Using the support matrix method, it is possible to improve the consistency of the measurement data of a single sensor, thereby reducing the influence abnormal measurement data have on the accuracy of the measurement system [23]. In this case, the single-sensor data fusion method is as follows: suppose that a sensor array contains m sensors of the same model, where *k* (*k* = 1, 2… A sensor, m) have *n* measurements, *X*_1_, *X*_2_, *X*_3_, …*X*_n_, respectively. Measured values *X_i_* to *X_j_* are provided to support available equations $R_{ij}=e^{-\left|X_{i}-X_{j}\right|}$. $R_{ij}=e^{-\left|X_{i}-X_{j}\right|}$, using exponential decay function quantitative sensor measurement data support, effectively solve the support value that, in traditional methods, can only take 1 or 0. The support matrix *R* between the N-measured values of the *K*th sensor is shown in Equation (6):

(6) $$R=\left|\begin{array}{cccc} R_{11} & R_{12} & \cdots & R_{1n}\\ R_{21} & R_{22} & \cdots & R_{2n}\\ \vdots & \vdots & \ddots & \vdots \\ R_{n1} & R_{n2} & \cdots & R_{nn} \end{array}\right|$$

The integrative supportability *a*(*i*) of the *i*th measured data of the sensor is shown in Equation (7):

(7) $$a(i)=\sum_{j=1}^{n}R_{ij}$$

If the integrative supportability of the measured data is large, it indicates that the consistency between the measured data and other measured data is higher, and that the weight ratio of the measured data is larger. A sensor is the first *k* of the measurement data of the weighted factor $W_{1k}(i)$, as shown in Equation (8):

(8) $$W_{1k}(i)=\frac{a(i)}{\sum_{i=1}^{n}a(i)}$$

Among them, $o\leq W_{1k}(i)\leq 1$, and it meets the $\sum_{i}^{n}W_{1k}(i)=1$. According to the support matrix method, the first n k sensor measurement value *X*_1_, *X*_2_, *X*_3_,…*X*_n_ weighting fusion estimate $\bar{X_{k}}$, as shown in Equation (9):

(9) $$\bar{X_{k}}=\sum_{i=1}^{n}W_{1k}(i)X_{i}$$

Upon obtaining the final weighted fusion estimation for a single sensor, the multi-sensor data fusion is carried out on the multi-group measurement signal sequences of the sensor array [24]. The adaptive weighted fusion estimation algorithm is used to estimate the fusion value of each sensor at a certain time and to find the corresponding optimal weighting factor in an adaptive way to minimize the total mean square error of the system [25].

After fusing the measured values of seven sensors in Figure 3, seven fusion estimates are obtained, as shown in Equation (10):

(10) $$X=\bar{X_{0}}*0.7+(\bar{X_{1}}+\bar{X_{2}}+\bar{X_{3}}+\bar{X_{4}}+\bar{X_{5}}+\bar{X_{6}}+\bar{X_{7}})*0.04286$$

where the *X*_0_ is sensor fusion estimation of the center and the rest are the estimations of outer-ring auxiliary sensors. The weight ratio of the central sensor 0.7 was selected as the coefficient after several tests.

The drunkenness level should be determined after the final fusion estimation, as shown in Table 2 [26]. Table 2 indicates that, if the blood alcohol concentration (BAC) reaches 20 mg/100 mL, the driver is considered to be intoxicated.

To accurately determine the level of drunk driving, the average deviation coefficient *β* is used, which is a parameter that is used to estimate the boundary between a set of average values in a given range [27]:

(11) $$\beta =[\sum_{i=0}^{i=n}\frac{\left|A_{i}-B\right|}{100}]*\frac{1}{n}$$

where *A_i_* is the measured value of the central sensor (0≤i≤n), *B* is the value of the grade line, and *N* is the number of measured values. First, the measured values of the central sensor are measured by the microcontroller for several times to take the average value. Second, the obtained average value is compared with the values of the seven drunk levels in Table 2. Finally, the average deviation coefficient of the data relative to the upper and lower boundaries is calculated by the drunk level interval of the average value according to Equation (11). By comparing the deviation coefficients, the level at which this group of data is more inclined to is determined, so as to show the drunkenness level and more visually alert the driver of their drunk driving status.

## 4. The Development of an Online Intelligent Detection Device for Drunk Driving of Automobile Drivers

### 4.1. Hardware Design

**Figure 4:**
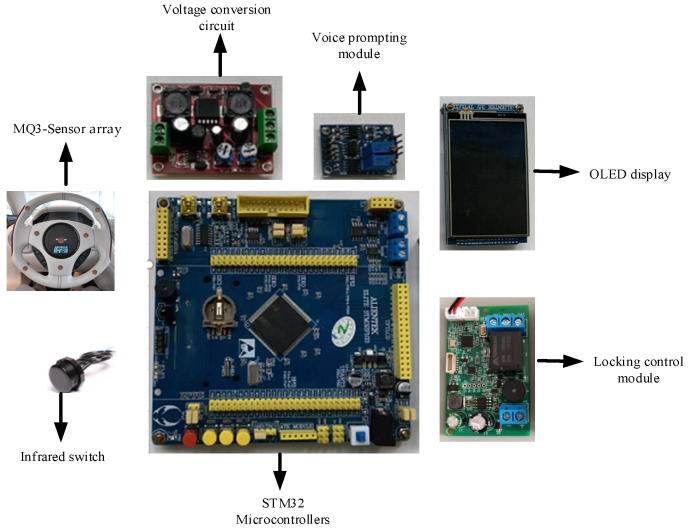
Hardware design of the device: STMicroelectronics manufactures the STM32 microcontroller, model number STM32F103ZET6; the MQ3 gas sensor is manufactured by YouXin Electronic, model number MQ3-1, and is made of tin dioxide; the infrared switch is manufactured by Xingkechuang, model number XKC-KD200; the voice chip manufacturer is Information Systems Development (ISD), model number ISD4004; the display is an OLED display of 1.3 inches, manufactured by Telesky; manufacturer of the relay is Tengfei, model number is JQC-T78-DC5V-C. The model number of ISD4004 is assigned by Information Systems Development; the display is an OLED display of 1.3 inches, manufactured by Telesky. The relay is manufactured by Tengfei and has the model number JQC-T78-DC5V-C.

The overall hardware design of the online intelligent detection device for the drunk driving of automobile drivers is shown in Figure 4. Its core circuit is composed of an STM32 microcontroller, an infrared switch, an MQ3 gas sensor array, an Qrganic Light-Emitting Diode (OLED) display screen, a voice alarm, a vehicle-started control relay, and a power supply and communication. When the infrared sensors detect the driver entered, the drunk driving detection system switches to the working state. Once the driver exhales air, the sensors detect the alcohol concentration. If it drunk driving is determined, the relay controls the ignition circuit in the car and disconnects it. At this point, the car cannot be started. The drunkenness level is then displayed on the screen.

### 4.2. Software Design

**Figure 5:**
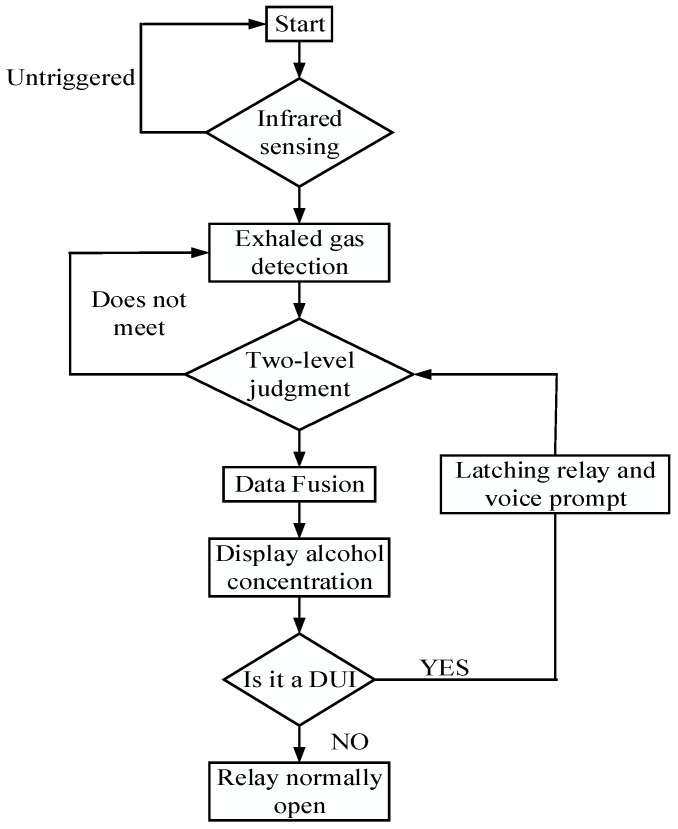
Device program flow.

According to the hardware design and data fusion algorithm, the device program flowchart, as shown in Figure 5, is formulated. The system workflow functions as follows: when the driver enters the car, their body irradiates an infrared ray to the infrared sensors, and the system collects the information and report it to the STM32 MCU, triggering the MQ3 sensors to collect information and determine whether the driver has been drinking from a two-level judgement system. If the driver has been drinking, it proceeds to data fusion, and the OLED display shows the alcohol concentration level and assesses the level of intoxication according to Table 2. Then, the relay is switched off, causing the car to fail to start; at the same time, the system automatically triggers the reminding device, and the microcontroller sends voice alarm module control information; next, the voice alarm module sends out an alarm to remind the driver that they are in a drunken state and should not drive a motor vehicle, as well as to please immediately shut off the throttle. If it does not reach a drunken driving level, the relays will open normally, and the vehicle can be normally started.

## 5. Test and Analysis

### 5.1. Sensor Array Testing

**Figure 6:**
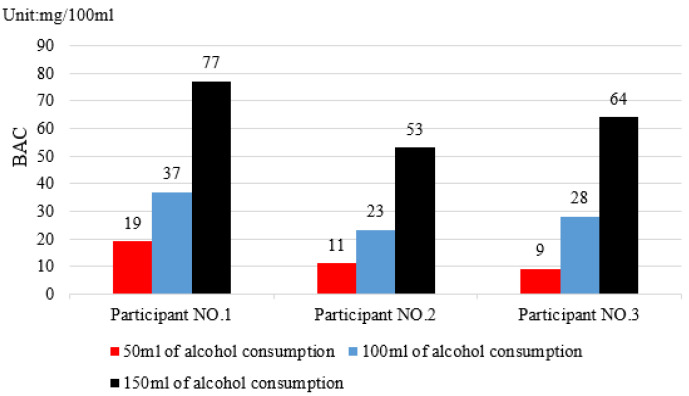
An illustration of the histograms of BAC (blood alcohol concentration) test data after one hour of drinking in subjects who consumed varying amounts of alcohol.

The first step is to test the accuracy of the detection of the sensor arrays based on a data fusion algorithm. Following the consumption of alcohol, three test participants breathed moderately on the MQ3 sensor array. The sensor output data and the voice alarm prompt. As a result of the actual driving situation of the driver, the breathing range of the subject from the sensor was set at 35 cm. The breath interval was 1 s. The measurement data represents the average value of five measurements taken within one minute. In this experiment, the first-class Baijiu was used with an alcohol content of 56% vol., and the standard number was GB/T 10781D. The physical conditions of the drivers are shown in Table 3. The test data for the different doses of blood alcohol concentration (BAC) values are presented in Figure 6.

Figure 6 shows the measured BAC values of the three participants 1 h after drinking alcohol; the test results indicated that, after drinking under 50 mL of alcohol, participant No. 1 had a BAC value of 19 mg/100 mL; participant No. 2 had a BAC value of 11 mg/100 mL; and participant No. 3 had a BAC value of 9 mg/100 mL. According to the judgment standard of driving under the influence (DUI), a person is considered intoxicated if the blood alcohol concentration exceeds 20 mg/100 mL. All three participants had BAC levels below 20 mg/100 mL; therefore, they were not considered as DUI. Therefore, the system cannot determine one’s DUI when less than 50 milliliters of alcohol has been consumed. After consuming 100 mL of alcohol, participant No. 1 had a blood alcohol concentration (BAC) of 37 mg/100 mL; participant No. 2 had a BAC of 23 mg/100 mL; and participant No. 3 had a BAC of 28 mg/100 mL. In the case of 150 mL of alcohol consumption, participant No. 1 had a BAC of 77 mg/100 mL; participant No. 2 had a BAC of 53 mg/100 mL; and participant No. 3 had a BAC of 64 mg/100 mL. Due to the consumption of 100 mL and 150 mL of alcohol, the BAC values exceeded 20 mg/100 mL, and all participants were determined to have been DUI. In addition, the experimental data revealed that the BAC values obtained from different individuals for the same drinking dose varied in response to factors such as body composition, metabolism, and the level of ethanol dehydrogenase synthesis [28]. Based on the results of the experiment, it was demonstrated that the sensor array was capable of detecting driving under the influence when the amount of alcohol consumed exceeded 50 milliliters.

### 5.2. Drunk Driving Practice Test

In order to verify the reliability of the first level judgment, to assess whether the drinker in the car is a driver or a non-driver, a simulation test was conducted according to the characteristics of the algorithm. In Experiment 1, the driver did not drink alcohol and the co-driver drank alcohol. The experimental environment was normal temperature, normal pressure, and no wind in the car. After drinking, subjects in the co-pilot position kept talking with the main driver and breathed towards the steering wheel with moderate strength. The experimental results are shown in Table 4.

According to the experimental results in Table 4, when the main driver has not consumed alcohol, that is, when the blood alcohol concentration is less than 3 mg/100 mL, and the co-driver has consumed alcohol, that is, when the blood alcohol concentration is more than 20 mg/100 mL, all the recognition results are shown as “input error”, indicating that the driver does not drink alcohol, and that there are people drinking in the car; however, there will be no misjudgment result.

To ensure that the system can accurately assess the driver’s drinking, Experiment 2 was carried out in an environment in which the driver had been drinking, the experimental environment was still under normal temperature, there was normal atmospheric pressure, and where there was no wind in the car. The three subjects had been drinking for a while and were in normal driving posture toward the steering wheel center, breathing with medium strength, and with a breathing time no less than 3 s. The experimental results are shown in Table 5.

According to the experimental results in Table 5, under the condition of 50 mL drinking dose, the alcohol content in the breath of the subject does not exceed 20 mg/100 mL, and the system does not consider it as drunk driving. Under the conditions of 100 mL and 150 mL, the BAC of the subject was all over 20 mg/100 mL, which was judged to be drunk driving. The experimental results show that the two-layer judgment system is reliable when the drinking amount of alcohol is more than 100 mL.

## 6. Conclusions

In order to solve the problem of error and easy misjudgment in the detection of automobile drunk driving by single sensor, this paper studies an online intelligent detection technology of automobile driver drunk driving based on multi-sensor data fusion technology, which has the features of a higher accuracy of detection results and low power consumption. Through the theoretical analysis and drunk driving test experiments, the following conclusions can be drawn:

(1) In order to obtain accurate detection results, the sensor array layout conforming to the drunk driving detection of the driver was studied. According to the gas turbulent diffusion model, the sensor array is located above the steering wheel, with one sensor in the center as the center sensor and the other six as auxiliary sensors;
(2) The multi-sensor data fusion model algorithm for drunk driving detection is proposed. The algorithm model of the multi-sensor data fusion model is divided into two layers. The first layer is assesses the driver driving under the influence of alcohol, so as to prevent misjudgment caused by non-driver drinking in the car. In the second layer, the degree of the alcohol consumption of the driver is detected based on the support matrix and the adaptive weighted fusion algorithm;
(3) The real-time online intelligent detection device of drunk driving has been constructed, and device simulation test results show that, based on the support and adaptive weighted sensor array data fusion system, we can improve the veracity and reliability of the intelligent test results of drunken driving. For a few unexpected situations, such as in the case where the drinker is not the driver, the system will shield from this situation and test again immediately. The system will not start until it detects that the driver is sitting in front of the cab, and the low-power standby mode is maintained, which greatly reduces the power consumption of the system;
(4) The experimental results indicate that DUI (driving under the influence) online intelligent detection can accurately determine DUI when the driver has consumed over 50 mL of alcohol and is sitting in the driver’s seat. However, drinking less than 50 mL of alcohol is not accurately detected as DUI, and the algorithm for data fusion will be further refined in the future to increase the accuracy of DUI detection. This paper has not yet considered the driver’s artificially caused DUI judgment errors, such as the use of a mask for testing, concealment of the MQ3 sensor, etc. It would be beneficial in the future to consider the use of multiple types of sensors and multi-information fusion to integrate anti-DUI devices for more accurate DUI judgments.

## Figures and Tables

**Table 1:** Description of ethanol diffusion in the car.

Position of Drunk Occupants	Car Environment	Detection Position	Diffusion Detection Results
Driving seat	No wind	Center and around the steering wheel	There is the greatest concentration on the steering wheel center and a decreasing concentration around it.
Co-pilot seat	No wind	Center and around the steering wheel	The concentration is highest near the right side (the co-pilot side) of the steering wheel and decreases from the center of the steering wheel to the left side of it.
Back seat	No wind	Center and around the steering wheel	There is a tendency to have a uniform concentration in the center and around the steering wheel.

**Table 2:** Level of drunkenness.

Level	BAC Value	Level	BAC Value
Level 1	20 mg/100 mL	Level 5	60 mg/100 mL
Level 2	30 mg/100 mL	Level 6	70 mg/100 mL
Level 3	40 mg/100 mL	Level 7	80 mg/100 mL
Level 4	50 mg/100 mL		

**Table 3:** Physical conditions of the test participants.

	Parameter	Height	Weight	Liver Function	Allergic to Alcohol	Have a History of Drinking
Serial Number						
Participant No. 1		170 cm	55 kg	Normal	No	Yes
Participant No. 2		176 cm	68 kg	Normal	No	Yes
Participant No. 3		182 cm	80 kg	Normal	No	Yes

**Table 4:** Judgment results of driver not drinking but co-driver drinking.

Main Driver Blood Alcohol Concentration	Co-Driver Blood Alcohol Concentration	Identification Results
<3 mg/100 mL	21 mg/100 mL	5 experiments, all blocked. The word “input error” is displayed
<3 mg/100 mL	37 mg/100 mL	5 experiments, all blocked. The word “input error” is displayed
<3 mg/100 mL	77 mg/100 mL	5 experiments, all blocked. The the word “input error” is displayed

**Table 5:** Driver drinking judgment results.

Alcohol Consumption	Time after Drinking	Judgment Results
50 mL	1 h	0 subjects had more than 20 mg/100 mL of alcohol in their breath.
100 mL	1 h	Three subjects had more than 20 mg/100 mL of alcohol in their breath.
150 mL	1 h	All three subjects had more than 20 mg/100 mL of alcohol in their breath, and one of them had more than 80 mg/100 mL.

## Data Availability

Not applicable.
